# Factors associated with serum CA125 level in women without ovarian cancer in the United States: a population-based study

**DOI:** 10.1186/s12885-022-09637-7

**Published:** 2022-05-14

**Authors:** Xiao Hu, Jingzhou Zhang, Yu Cao

**Affiliations:** 1 Division of Hematology-Oncology, Department of Medicine, Tufts Medical Center, Boston, MA USA; 2 Department of Medicine, Boston University School of Medicine, Boston, MA USA

**Keywords:** CA125, Epidemiology, Ovarian cancer

## Abstract

**Background:**

Cancer antigen 125 (CA125) is clinically used to monitor response to therapy in ovarian cancer and has been proposed for use in detecting ovarian cancer. This population-based study examines how demographic characteristics, gynecologic/reproductive history, chronic non-malignant medical conditions, history of non-ovarian cancer, lifestyle practices, and biomarkers of inflammation correlate with serum CA125 in both premenopausal and postmenopausal women without ovarian cancer across the United States.

**Methods:**

Participants were identified from the National Health and Nutrition Examination Survey 2001–2002. Linear and logistic regression models were applied.

**Results:**

Higher CA125 levels were found to correlate with younger age, Non-Hispanic White race/ethnicity, and lower body mass index. In premenopausal women (*N* = 1157), current smoking was associated with lower CA125 (− 24.95%, *p* = 0.008), and history of non-ovarian cancer was associated with higher CA125 (40.64%, *p* = 0.045) by multivariable linear regression; both current smoking (odds ratio (OR) = 0.42, *p* = 0.043) and oral contraceptive pill (OCP) use of 5–10 years (OR = 0.31, *p* = 0.032) were less likely to be associated with having CA125 level ≥ 35 U/ml by multivariable logistic regression. In postmenopausal women (*N* = 1116), coronary artery disease (CAD) history was associated with higher CA125 (28.27%, *p* = 0.047) by multivariable linear regression; history of CAD (OR = 5.00, *p* = 0.011), history of breastfeeding (OR = 2.46, *p* = 0.026), and increased CRP level (OR = 1.41, *p* = 0.042) were more likely to be associated with having CA125 level ≥ 35 U/ml by multivariable logistic regression.

**Conclusions:**

Results suggest CA125 is lower in premenopausal women who are current smokers and OCP users of moderately longer duration but higher in those with non-ovarian cancer. CA125 is higher in those postmenopausal women with CAD, history of breastfeeding and elevated CRP level. These associations can inform clinical interpretation of individual patients’ CA125 levels.

**Supplementary Information:**

The online version contains supplementary material available at 10.1186/s12885-022-09637-7.

## Background

Ovarian cancer (OC) is the deadliest of all gynecologic cancers, contributing to 5% of estimated cancer mortality among women in the United States in 2020 [1]. As OC tends to present in advanced stage, the need for more effective screening methods and biomarkers for early detection remains urgent. Cancer antigen 125 (CA125) is a tumor marker that is elevated in over 80% of patients with epithelial OC [2]. CA125 is a high molecular weight glycoprotein, also known as mucin 16, encoded by *MUC16* and recognized by the murine monoclonal antibody OC125 [2, 3]. Serial measurements of serum CA125 levels are clinically useful for monitoring response to chemotherapy in OC and may be a prognostic indicator [4, 5]. Current guidelines do not support the use of CA125 for population-based screening for OC in asymptomatic women, as randomized controlled trials have not demonstrated mortality reduction [6, 7].

Given that mucin 16 has wide physiologic expression throughout body cavities, in tissues lining the aerodigestive and female reproductive tracts, and in organs including gallbladder and eyes, serum CA125 levels could be affected by myriad benign and malignant processes [8]. Chronic benign medical conditions including endometriosis, fibroids, and those associated with ascites or inflammation such as heart failure, liver cirrhosis and ulcerative colitis, have been reported to be associated with higher CA125 levels, while osteoporosis, osteoarthritis, and hyperlipidemia have been reported to be associated with lower CA125 levels [8–10]. In addition to OC, elevated CA125 levels have been reported in breast, uterine, non-small cell lung, pancreatic, stomach and liver cancers [8, 11–16]. Furthermore, demographic characteristics such as age and race, menopausal status, gravidity/parity, history of gynecologic surgery, menstrual phase at time of blood test, oral contraceptive pill (OCP) use, receipt of hormone replacement therapy (HRT), and lifestyle factors such as smoking history and caffeine consumption may all influence CA125 levels [9, 10, 17].

With the limited sensitivity and specificity of CA125, efforts are ongoing to develop better screening biomarkers for OC as well as to elucidate how CA125 levels may be optimally utilized and interpreted in individual patients. Previous studies examining various factors correlating with CA125 levels were largely conducted in postmenopausal women extracted from control groups in existing clinical trials, and geographically limited to Europe and certain regions of the United States, particularly the Northeast (Table 1) [9, 10, 17]. We therefore conducted a population-based study to examine factors associated with CA125 level in both premenopausal and postmenopausal women without OC across the United States.

**Table 1:** Summary of studies examining CA125 level in women without ovarian cancer

Authors (Year)	Number of patients	Menopausal status	CA125 assay used	Association with higher CA125	Association with lower CA125	Reference number
Pauler et al. (2001)	18,748 (St. Bartholomew’s/ Royal London Hospital Ovarian Cancer screening trial)	Postmenopausal	CA125II radioimmunoassay (Centocor) at a single laboratory site	-Caucasian race -Prior cancer diagnosis other than ovarian cancer	-Age -African/Asian races -Hysterectomy -Regular smoking -Regular caffeine consumption	[17]
Johnson et al. (2008)	25,608 (PLCO study)	Postmenopausal	CA125II radioimmunoassay (Centocor)	-Age -Former smoking -Ever use of hormone therapy -History of breast cancer	-Non-White status -Current smoking -Hysterectomy -Obesity	[18]
Akinwunmi et al. (2018)	2,004 (NEC study)	Premenopausal, Postmenopausal	CA125II radioimmunoassay, Clinical and Epidemiology Research Laboratory (CERLab) at Boston Children’s Hospital	*Premenopausal:* -Endometriosis -Coronary artery disease	*Premenopausal:* -Hysterectomy -Colon polyps	[9]
				*Postmenopausal:* -Inflammatory bowel disease	*Postmenopausal:* -Osteoporosis -Osteoarthritis -Hypercholesterolemia	
Sasamoto et al. (2019)	815 (NEC study, model development) 473 (EPIC study, model validation)	Premenopausal	CA125II radioimmmunoassay (Centocor, Malvern, PA), CERLab at Boston Children’s Hospital (NEC study) Meso Scale Discovery (MSD): volume-effective highly sensitive multiplex platform (Gaithersburg, MD), Genital Tract Biology Laboratory, Brigham and Women’s Hospital (EPIC study)	-Age 30–39 -Early follicular menstrual phase -Endometriosis -Fibroids	-Age < 30 or > 50 -Menstrual phases other than early follicular -Current hormonal contraception use -Tubal ligation	[19]
Sasamoto et al. (2019)	26,981 (PLCO study, model development) 861 (EPIC study) + 81 (NHS/NHSI) + 923 (NEC study) (model validation)	Postmenopausal	CA-125II radioimmunoassay (Centocor) (PLCO study) CA-125II radioimmunoassay (Centocor), CERLab at Boston Children’s Hospital (NEC and NHS/NHSII studies) Meso Scale Discovery (MSD): volume-effective highly sensitive multiplex platform (Gaithersburg, MD), Genital Tract Biology Laboratory, Brigham and Women’s Hospital (EPIC study)	-Age -White race -Lower BMI -Former smoking status -Shorter duration of smoking among former smokers -Older age at first menstrual period -Older age at last menstrual period -Shorter time since menopause -Higher parity -History of benign ovarian cyst -Ever use and longer duration of hormone therapy	-Hysterectomy	[10]

## Materials and methods

### Data source

Women without OC were identified from the National Health and Nutrition Examination Survey (NHANES) 2001–2002, a nationally representative, cross-sectional survey conducted in the non-institutionalized, civilian United States population using a complex, multistage, probability sampling design [20]. NHANES collected demographic, nutritional, and health condition data of the survey participants, and CA125 data were measured in NHANES 2001–2002. Women with OC or without CA125 measurements were excluded. All participants provided written informed consent, and the survey protocol was approved by the NCHS (National Center for Health Statistics) Research Ethics Review Board.

### Menopausal status

Premenopausal status was defined as having regular periods in the past 12 months before interview or not having regular periods due to current or recent pregnancy, breastfeeding, having irregular periods at baseline, or other medical conditions. Women who did not have regular periods in the past 12 months due to going or having gone through menopause, or those who had hysterectomy were classified as postmenopausal.

### CA125 measurement

Serum samples of female participants with age ≥ 20 years who had stored samples from 2001 to 2002 were used for CA125 measurements, performed in the Genital Tract Biology Laboratory (Brigham and Women’s Hospital, Boston, MA) following pre-analytic, analytic, and post-analytic standardized operational procedures. Meso Scale Discovery electrochemiluminescence immunoassay platform was used for the measurements. A coefficient of variation (CV) of < 25% was set as a threshold for acceptance of the data. CA125 measurements were reported as U/mL [21].

### Exposure variables

We focused on examining factors which have been reported in at least one previous study to be significantly associated with CA125 level, [9, 17, 18, 22] and included factors which have been studied more sporadically however are biologically plausible to be associated with CA125 [8, 9, 17]. These factors included demographics (age, race/ethnicity), body mass index (BMI, kg/m^2^), reproductive history (age at menarche, parity, breastfeeding history, current hormonal contraception (CHC) use, OCP use and duration of use), HRT (exclusively in postmenopausal women), prior gynecologic surgery such as history of hysterectomy (exclusively in postmenopausal women), oophorectomy (exclusively in postmenopausal women), salpingectomy, lifestyle practices (smoking/tobacco use status, alcohol consumption, caffeine intake), history of chronic non-malignant medical conditions (endometriosis, fibroids, osteoporosis, osteoarthritis, coronary artery disease (CAD), hyperlipidemia), history of non-ovarian cancer, and laboratory measurements of biomarkers of inflammation (C reactive protein (CRP), ferritin).

Demographic data were collected at interview. BMI data were obtained from the high-quality body measurements performed on survey participants at exam visit. All reproductive health history was collected via face-to-face interview. CHC use was only assessed in premenopausal women and was defined as taking OCP now or using Depo-Provera or injectables now to prevent pregnancy; information regarding the use of intrauterine device with hormones was not collected in NHANES 2001–2002. Current smoking was defined as smoking cigarettes daily or some days now by self-report, or with a serum nicotine level > 15 ng/ml [23]. Alcohol consumption was categorized as “non-drinking”, “one to two drinks per day” and “more than two drinks per day” according to average drinks per day in the past 12 months before the interview. The amount of caffeine intake (mg) was estimated based on the participants’ dietary recall during the 24-hour period prior to the interview. Hysterectomy, oophorectomy, salpingectomy, and other chronic medical conditions were defined as ever having this procedure or condition by self-reported personal interview data. Cancer history was per participants’ self-report in reply to questions of “ever told you had cancer or malignancy” and “what kind of cancer” in the survey. Serum CRP level (mg/dL) was measured by latex-enhanced nephelometry and serum ferritin level (ng/mL) was determined by the STA-Compact via the Clauss clotting method.

### Statistical analysis

The distribution of CA125 measurements across four quantiles was outlined for all candidate correlating factors. CA125 levels were transformed logarithmically to be more normally distributed. Univariable and multivariable linear regressions were used to examine associations between factors of interest and CA125 levels in premenopausal and postmenopausal subgroups. Additionally, CA125 levels were dichotomized per a cut-off value of 35 U/ml (< 35 U/ml as reference), which is the conventionally used upper limit of normal for CA125 derived from prior studies [2, 24, 25]. Univariable and multivariable logistic regressions were conducted to examine factors associated with CA125 levels ≥35 U/ml. Age, BMI, caffeine intake, CRP, and ferritin levels were entered as continuous variables. Race/ethnicity, parity, breastfeeding history, CHC use, OCP use, menopausal status, current HRT use status, history of hysterectomy, oophorectomy, salpingectomy, smoking status, alcohol use status, history of endometriosis, fibroids, osteoporosis, osteoarthritis, CAD, hyperlipidemia, and history of non-ovarian cancer were treated as categorical variables. Factors known to correlate with CA125 (age, race/ethnicity, BMI, parity) were adjusted for in all subgroup analyses. For postmenopausal women, current HRT use was additionally adjusted for in regression analyses. For all crude or univariable analyses, any variable with a *p* value < 0.25 was included in the multivariable model. Two-sided *p* values < 0.05 were considered statistically significant for multivariable analysis. We used *PROC SURVEY* in SAS to account for the complex sampling design of NHANES. SAS 9.4 (SAS Institute, Cary, NC) and R 3.6.2 (R Foundation for Statistical Computing, Vienna, Austria) were used.

## Results

A total of 2476 women with available CA125 measurements were finally included and analyzed. In this sample, subjects’ ages were between 20 to 85 years with a median age of 45 years. Majority of participants were Non-Hispanic White (52.8%), followed by 20.9% Mexican American participants, 18.1% Non-Hispanic Black participants, 4.4% other Hispanic participants, and 3.8% participants representing other races. The demographic characteristics and distribution of CA125 levels across four quantile ranges demonstrated that higher CA125 levels appeared to correlate with younger age, Non-Hispanic White race/ethnicity, and lower BMI (Table 2).

**Table 2:** Distribution of CA125 measurements by candidate correlating factors across four quantile ranges (Q1 = [0.7300, 7.6575], Q2 = [7.6575, 11.6050], Q3 = [11.6050, 18.7750], Q4 = [18.7750, 569.37] U/ml)

Variables	Q1 CA125 (***N*** = 619)	Q2 CA125 (***N =*** 619)	Q3 CA125 (***N =*** 619)	Q4 CA125 (***N =*** 618)	Overall (***N*** = 2476)
**Race/Ethnicity**					
Non-Hispanic White	293 (47.3%)	313 (50.6%)	331 (53.5%)	370 (59.9%)	1307 (52.8%)
Non-Hispanic Black	156 (25.2%)	116 (18.7%)	87 (14.1%)	89 (14.4%)	448 (18.1%)
Mexican American	114 (18.4%)	143 (23.1%)	149 (24.1%)	112 (18.1%)	518 (20.9%)
Other Hispanic	37 (6.0%)	19 (3.1%)	25 (4.0%)	26 (4.2%)	108 (4.4%)
Other	19 (3.1%)	28 (4.5%)	27 (4.4%)	21 (3.4%)	95 (3.8%)
**Age (years)**					
Mean (SD)	52.9 (18.2)	49.2 (19.1)	46.2 (19.3)	44.1 (19.4)	48.1 (19.3)
Median [Min, Max]	53.0 [20.0, 85.0]	49.0 [20.0, 85.0]	42.0 [20.0, 85.0]	38.0 [20.0, 85.0]	45.0 [20.0, 85.0]
**BMI (kg/m**^**2**^**)**					
Mean (SD)	29.1 (7.32)	28.8 (6.67)	28.2 (6.61)	27.7 (6.46)	28.5 (6.79)
Median [Min, Max]	27.6 [15.8, 65.4]	27.7 [16.2, 55.4]	27.3 [17.0, 59.9]	26.5 [16.4, 61.7]	27.3 [15.8, 65.4]
**Smoking history**					
Non-smoker	361 (58.3%)	391 (63.2%)	384 (62.0%)	378 (61.2%)	1514 (61.1%)
Former smoker	132 (21.3%)	113 (18.3%)	136 (22.0%)	126 (20.4%)	508 (20.5%)
Current smoker	126 (20.4%)	115 (18.6%)	99 (16.0%)	114 (18.4%)	454 (18.3%)
**Alcohol use history**					
Non-drinking	198 (37.8%)	206 (38.7%)	205 (38.5%)	195 (37.7%)	805 (38.2%)
One-two drinks per day	252 (48.1%)	243 (45.7%)	250 (47.0%)	242 (46.7%)	987 (46.8%)
More than two drinks per day	74 (14.1%)	83 (15.6%)	77 (14.5%)	81 (15.6%)	315 (15.0%)
**Daily caffeine intake (mg)**					
Mean (SD)	133 (178)	125 (157)	119 (153)	124 (155)	125 (161)
Median [Min, Max]	83.0 [0.00, 1920]	77.0 [0.00, 1140]	72.0 [0.00, 1490]	74.0 [0.00, 1190]	77.0 [0.00, 1920]
**Age at menarche (years)**					
≤ 12	267 (47.8%)	266 (48.3%)	250 (44.2%)	253 (47.0%)	1036 (47.8%)
> 12	292 (52.2%)	285 (51.7%)	315 (55.8%)	285 (53.0%)	1178 (53.2%)
**Parity**					
None	16 (3.2%)	11 (2.3%)	32 (6.5%)	21 (4.4%)	80 (4.1%)
One	76 (15.4%)	86 (17.8%)	108 (21.9%)	113 (23.7%)	383 (19.7%)
Two	143 (29.0%)	133 (27.5%)	127 (25.7%)	150 (31.5%)	553 (28.4%)
Three	103 (20.9%)	125 (25.8%)	107 (21.7%)	105 (22.1%)	440 (22.6%)
Four or more	155 (31.4%)	129 (26.7%)	120 (24.3%)	87 (18.3%)	492 (25.3%)
**Breastfeeding history**					
No	205 (43.6%)	180 (39.1%)	165 (36.7%)	161 (36.9%)	711 (39.1%)
Yes	265 (56.4%)	280 (60.9%)	285 (63.3%)	275 (63.1%)	1106 (60.9%)
**Menopause**					
No	203 (35.6%)	252 (44.3%)	338 (58.6%)	364 (65.6%)	1157 (50.9%)
Yes	368 (64.4%)	317 (55.7%)	239 (41.4%)	191 (34.4%)	1116 (49.1%)
**Duration of oral contraceptive use (years)**					
Less than 2 years	172 (49.3%)	154 (44.8%)	144 (41.1%)	153 (42.5%)	623 (44.4%)
2–5 years	71 (20.3%)	72 (20.9%)	80 (22.9%)	99 (27.5%)	322 (23.0%)
5–10 years	68 (19.5%)	72 (20.9%)	82 (23.4%)	74 (20.6%)	296 (21.1%)
More than 10 years	38 (10.9%)	46 (13.4%)	44 (12.6%)	34 (9.4%)	162 (11.5%)
**Current hormonal contraception use**					
No	18 (25.7%)	15 (23.4%)	22 (31.4%)	16 (34.8%)	71 (28.4%)
Yes	52 (74.3%)	49 (76.6%)	48 (68.6%)	30 (65.2%)	179 (71.6%)
**Current hormone replacement therapy**					
No	521 (84.2%)	522 (84.3%)	558 (90.1%)	570 (92.2%)	2172 (87.7%)
Yes	98 (15.8%)	97 (15.7%)	61 (9.9%)	48 (7.8%)	304 (12.3%)
**C reactive protein level (mg/dL)**					
Mean (SD)	0.540 (0.888)	0.554 (0.824)	0.549 (1.030)	0.622 (1.090)	0.566 (0.962)
Median [Min, Max]	0.290 [0.010, 14.0]	0.290 [0.010, 10.7]	0.280 [0.010, 18.5]	0.330 [0.010, 16.3]	0.300 [0.010, 18.5]
**Ferritin level (ng/mL)**					
Mean (SD)	93.5 (107)	79.1 (99.6)	66.7 (87.2)	67.9 (99.1)	77.0 (99.8)
Median [Min, Max]	61.0 [3.0, 1040]	47.0 [3.0, 1230]	39.0 [3.0, 933]	33.0 [3.0, 981]	44.0 [3.0, 1230]
**History of hysterectomy**					
No	244 (55.5%)	237 (58.8%)	234 (70.5%)	264 (76.5%)	979 (64.4%)
Yes	196 (44.5%)	166 (41.2%)	98 (29.5%)	81 (23.5%)	542 (35.6%)
**History of oophorectomy**					
No	423 (75.0%)	462 (81.5%)	505 (87.8%)	493 (89.3%)	1883 (83.4%)
Yes	141 (25.0%)	105 (18.5%)	70 (12.2%)	59 (10.7%)	376 (16.6%)
**History of salpingectomy**					
No	410 (72.3%)	429 (75.9%)	448 (77.8%)	446 (80.8%)	1734 (76.7%)
Yes	157 (27.7%)	136 (24.1%)	128 (22.2%)	106 (19.2%)	527 (23.3%)
**History of endometriosis**					
No	263 (90.4%)	313 (94.0%)	370 (59.8%)	367 (91.5%)	1313 (92.9%)
Yes	28 (9.6%)	20 (6.0%)	18 (2.9%)	34 (8.5%)	100 (7.1%)
**History of uterine fibroids**					
No	246 (84.5%)	295 (88.9%)	345 (89.4%)	361 (89.6%)	1247 (88.3%)
Yes	45 (15.5%)	37 (11.1%)	41 (10.6%)	42 (10.4%)	165 (11.7%)
**History of osteoporosis**					
No	554 (90.1%)	554 (89.8%)	562 (91.5%)	568 (92.2%)	2238 (90.9%)
Yes	61 (9.9%)	63 (10.2%)	52 (8.5%)	48 (7.8%)	225 (9.1%)
**History of osteoarthritis**					
No	566 (91.4%)	557 (90.0%)	569 (91.9%)	571 (92.4%)	2264 (91.4%)
Yes	53 (8.6%)	62 (10.0%)	50 (8.1%)	47 (7.6%)	212 (8.6%)
**History of coronary artery disease**					
No	593 (96.3%)	601 (97.4%)	604 (97.9%)	595 (97.4%)	2394 (97.2%)
Yes	23 (3.7%)	16 (2.6%)	13 (2.1%)	16 (2.6%)	68 (2.8%)
**History of hyperlipidemia**					
No	269 (61.3%)	255 (61.3%)	270 (66.3%)	278 (68.6%)	1072 (64.3%)
Yes	170 (38.7%)	161 (38.7%)	137 (33.7%)	127 (31.4%)	595 (35.7%)
**History of non-ovarian cancer**					
No	564 (91.1%)	567 (91.6%)	557 (90.3%)	573 (93.2%)	2262 (91.5%)
Yes	55 (8.9%)	52 (8.4%)	60 (9.7%)	42 (6.8%)	209 (8.5%)

*SD * Standard deviation, *BMI* Body mass index

Menopausal status was available for all except 203 participants who were excluded from subgroup analyses requiring menopausal status. Of 2273 women with available menopausal status, 49.1% reported to have postmenopausal status. The mean CA125 levels of premenopausal and postmenopausal subgroups were 18.02 U/ml (standard deviation (SD), 16.63 U/ml) and 13.53 U/ml (SD 21.52 U/ml), respectively. A total of 158 (6.38%) women had a CA125 level ≥ 35 U/ml.

### Premenopausal subgroup

For premenopausal women (*N* = 1157), current smoking, breastfeeding history, CHC use, CRP level, history of osteoporosis, osteoarthritis, salpingectomy, endometriosis, fibroids, CAD, and non-ovarian cancer demonstrated possible associations with CA125 concentration (*p* < 0.25) via linear regression models (Table 3). However, given the low frequency of osteoporosis (*N* = 13), osteoarthritis (*N* = 30) and CAD (*N* = 10) in the premenopausal subgroup, these factors were not included in the final multivariable analysis. In multivariable linear regression, current smoking status and history of non-ovarian cancer remained significantly associated with CA125 level. Compared to non-smokers, premenopausal current smokers (*N* = 256) have a lower mean CA125 level (− 24.95%, 95% confidence interval [CI] [− 37.44%, − 9.97%], *p* = 0.008). Premenopausal women with history of non-ovarian cancer (*N* = 37) have much higher CA125 levels than those without this history (40.64%, 95% CI [3.56%, 90.98%], *p* = 0.045).

**Table 3:** Associations between candidate correlating factors and percent change in CA125 levels among premenopausal women (*N =* 1157) by univariable and multivariable linear regressions

Variables	Univariable linear regressions^**a**^			Multivariable linear regressions^**b**^			
	Effect estimate	CA125 change	*P* value	Effect estimate	Standard error	CA125 change	*P* value
**Smoking**							
Current^d^	−0.124	−11.66%	0.047	−0.287	0.093	−24.95%	**0.008**
Former	0.065	6.72%	0.330	0.014	0.194	1.41%	0.943
None	ref						
**EtOH**							
> 2 drinks/day	−0.049	−4.78%	0.653				
1–2 drinks/day	0.002	0.20%	0.972				
None	ref						
**Caffeine**	−0.0001	−0.01%	0.404				
**Age of menarche**							
> 12	0.039	3.98%	0.479				
≤ 12	ref						
**Breastfeed**^**c**^	0.093	9.75%	0.078	0.110	0.121	11.63%	0.378
**CHC use**	−0.155	−14.36%	0.218	−0.108	0.108	−10.24%	0.337
**OCP duration**							
10+ years	−0.111	−10.51%	0.310				
5–10 years	0.057	5.87%	0.502				
2–5 years	0.113	11.96%	0.278				
< 2 years	ref						
**CRP**	0.061	6.29%	0.138	0.041	0.078	4.19%	0.607
**Ferritin**	−0.0003	− 0.03%	0.453				
**Osteoporosis**	−0.373	−31.13%	0.049				
**Osteoarthritis**	0.312	36.62%	0.067				
**Salpingectomy**	−0.121	−11.40%	0.092	0.132	0.194	14.11%	0.506
**Endometriosis**	0.234	26.36%	0.115	0.057	0.271	5.87%	0.837
**Fibroids**	0.153	16.53%	0.184	−0.130	0.171	−12.19%	0.460
**CAD**	−0.689	−49.79%	0.001				
**HLD**	−0.037	−3.63%	0.700				
**Non-ovarian cancer**^d^	0.216	24.11%	0.106	0.341	0.156	40.64%	**0.045**

*EtOH* Alcohol, *CHC* Current hormonal contraception, *OCP* Oral contraceptive pill, *CRP* C reactive protein, *CAD* Coronary artery disease, *HLD* Hyperlipidemia

^**a**^ All univariable regressions were adjusted for baseline variables including age, race/ethnicity, BMI, and parity

^**b**^ Multivariable regressions included the baseline variables (age, race/ethnicity, BMI, parity) and eligible variables from univariable analyses with cut-off *p* value < 0.25

^c^ For the “Breastfeed” variable, the reference for parity is one instead of none

^d^ These variables demonstrated significant associations with CA125 levels in the multivariable linear regression model

Univariable logistic regressions showed that among premenopausal women, current smoking, OCP use for 2–5 years and for 5–10 years, CRP level, and history of endometriosis demonstrated possible associations (*p* < 0.25) with CA125 levels ≥ 35 U/ml (*N* = 101) (Table 4). Of note, given the low frequency of osteoporosis (*N* = 13, and only 1 had CA125 level ≥ 35 U/ml), osteoarthritis (*N* = 30, and only 3 had CA125 level ≥ 35 U/ml) and CAD (*N =* 10, but none had CA125 levels ≥ 35 U/ml) in the premenopausal subgroup, these variables were excluded from these analyses. Multivariable logistic regression analysis identified that current smoking and OCP use for 5–10 years, still demonstrated significant associations. Compared with no smoking, current smoking (*N* = 256) in premenopausal women was less likely to be associated with CA125 concentration ≥ 35 U/ml (odds ratio (OR) = 0.42, 95% CI [0.18–0.97], *p* = 0.043). Compared to a short duration of OCP use (less than 2 years), OCP use of 5–10 years (*N* = 178) in premenopausal women was less likely to be associated with CA125 level ≥ 35 U/ml (OR = 0.31, 95% CI [0.11–0.91], *p* = 0.032).

**Table 4:** Associations between candidate correlating factors and CA125 level ≥ 35 U/ml among premenopausal women (*N =* 1157) by univariable and multivariable logistic regressions

Variables	Univariable logistic regressions^**a**^				Multivariable logistic regressions^**b**^			
	Effect estimate	Odds ratio (OR)	*P* value	Effect estimate	Standard error	OR	*P* value	
**Smoking**								
Current^d^	−0.654	0.520	0.0893	−0.863	0.426	0.422	**0.043**	
Former	0.311	1.365	0.471	0.579	0.400	1.784	0.148	
None	ref							
**EtOH**								
> 2 drinks/day	−0.165	0.848	0.679					
1–2 drinks/day	0.092	1.096	0.763					
None	ref							
**Caffeine**	−0.00037	1.000	0.530					
**Age of menarche**								
> 12	0.118	1.125	0.634					
≤ 12	ref							
**Breastfeed**^**c**^	0.012	1.012	0.968					
**CHC use**	−1.019	0.361	0.570					
**OCP duration**								
10+ years	−0.483	0.617	0.407	−0.596	0.621	0.551	0.337	
5–10 years^d^	− 0.967	0.380	0.098	−1.173	0.548	0.309	**0.032**	
2–5 years	0.528	1.695	0.234	0.465	0.474	1.592	0.327	
< 2 years	ref							
**CRP**	0.325	1.383	0.154	0.295	0.260	1.343	0.256	
**Ferritin**	−0.00195	0.998	0.621					
**Salpingectomy**	−0.616	0.540	0.278					
**Endometriosis**	0.857	2.355	0.029	0.763	0.631	2.145	0.226	
**Fibroids**	0.456	1.578	0.468					
**HLD**	−0.412	0.662	0.480					
**Non-ovarian cancer**	0.058	1.060	0.938					

*EtOH* Alcohol, *CHC* Current hormonal contraception, *OCP* Oral contraceptive pill, *CRP* C reactive protein, *CAD* Coronary artery disease, *HLD* Hyperlipidemia

^a^ All univariable regressions were adjusted for baseline variables including age, race/ethnicity, BMI and parity

^b^ Multivariable regressions included the baseline variables (age, race/ethnicity, BMI, parity) and eligible variables from univariable analyses with cut-off *p* value < 0.25

^c^ For the “Breastfeed” variable, the reference for parity is one instead of none

^d^ These variables demonstrated significant associations with CA125 level ≥ 35 U/ml in the multivariable logistic regression model

### Postmenopausal subgroup

For postmenopausal women (*N* = 1116), alcohol consumption of more than two drinks per day, caffeine intake, CRP, ferritin level, history of hysterectomy, oophorectomy, endometriosis, fibroids, and CAD demonstrated possible associations with CA125 concentration (*p* < 0.25) via linear regression models (Table 5). In multivariable linear regression analysis, only history of CAD remained significantly associated with CA125 level. Postmenopausal women with history of CAD (*N* = 58) have higher CA125 levels than those without this history (28.27%, 95% CI [2.39%, 60.64%], *p* = 0.047).

**Table 5:** Associations between candidate correlating factors and percent change in CA125 levels among postmenopausal women (*N =* 1116) by univariable and multivariable linear regressions

Variables	Univariable linear regressions^**a**^			Multivariable linear regressions^**b**^			
	Effect estimate	CA125 change	*P* value	Effect estimate	Standard error	CA125 change	*P* value
**Smoking**							
Current	−0.096	−9.15%	0.258				
Former	−0.014	−1.39%	0.835				
None	ref						
**EtOH**							
> 2 drinks/day	−0.178	−16.31%	0.076	−0.267	0.129	−23.43%	0.055
1–2 drinks/day	0.021	2.12%	0.545	0.050	0.072	5.13%	0.499
None	ref						
**Caffeine**	−0.0002	−0.02%	0.034	−0.00002	0.0002	−0.002%	0.887
**Age of menarche**							
> 12	0.020	2.02%	0.687				
≤ 12	ref						
**Breastfeed**^**c**^	0.020	2.02%	0.689				
**OCP duration**							
10+ years	0.009	0.90%	0.914				
5–10 years	−0.002	−0.20%	0.978				
2–5 years	0.070	7.25%	0.543				
< 2 years	ref						
**CRP**	0.057	5.87%	0.074	−0.028	0.069	−2.76%	0.696
**Ferritin**	−0.0003	−0.03%	0.138	0.00062	0.0006	0.06%	0.473
**Osteoporosis**	−0.017	−1.69%	0.750				
**Osteoarthritis**	−0.048	−4.69%	0.333				
**Hysterectomy**	−0.084	−8.06%	0.050	−0.095	0.120	−9.06%	0.441
**Oophorectomy**	−0.104	−9.88%	0.061	−0.050	0.102	−4.88%	0.628
**Salpingectomy**	0.044	4.50%	0.381				
**Endometriosis**	−0.205	−18.54%	0.126	−0.068	0.112	−6.57%	0.554
**Fibroids**	−0.242	−21.49%	0.046	−0.161	0.132	−14.87%	0.242
**CAD**^**d**^	0.131	14.00%	0.134	0.249	0.115	28.27%	**0.047**
**HLD**	0.054	5.55%	0.364				
**Non-ovarian cancer**	−0.048	−4.69%	0.299				

*EtOH* alcohol, *CHC* current hormonal contraception, *OCP* oral contraceptive pill, *CRP* C reactive protein, *CAD* coronary artery disease, *HLD* hyperlipidemia

^a^ All univariable regressions were adjusted for baseline variables including age, race/ethnicity, BMI, parity and current hormonal replacement therapy (HRT) use

^b^ Multivariable regressions included the baseline variables (age, race/ethnicity, BMI, parity, HRT) and eligible variables from univariable analyses with cut-off *p* value < 0.25

^**c**^ For the “Breastfeed” variable, the reference for parity is one instead of none

^d^ This variable demonstrated significant association with CA125 level in the multivariable linear regression model

Univariable logistic regressions showed that among postmenopausal women, breastfeeding history, CRP level, CAD, and non-ovarian cancer demonstrated possible associations with CA125 levels ≥ 35 U/ml (*N* = 41) (*p* < 0.25) (Table 6). Of note, as only a limited number of postmenopausal participants who drank alcohol had CA125 level ≥ 35 U/ml (*N* = 23 out of 556), and no postmenopausal women with endometriosis had CA125 level ≥ 35 U/ml, no crude logistic regression was performed with respect to alcohol consumption or endometriosis. After performing multivariable logistic regression, breastfeeding history, CRP, and CAD remained significant. History of breastfeeding in postmenopausal women (*N* = 561) was more likely to be associated with CA125 level ≥ 35 U/ml (OR = 2.46, 95% CI [1.12, 5.41] *p* = 0.026), as was history of CAD (*N* = 58) (OR = 5.00, 95% CI [1.45, 17.17], *p* = 0.011). Additionally, for a one-unit increase in CRP level, there was 41.10% of increase in the odds of having CA125 ≥ 35 U/ml (OR = 1.41, 95% CI [1.01, 1.97], *p* = 0.042).

**Table 6:** Associations between candidate correlating factors and CA125 level ≥ 35 U/ml among postmenopausal women (*N =* 1116) by univariable and multivariable logistic regressions

Variables	Univariable logistic regressions^**a**^			Multivariable logistic regressions^**b**^			
	Effect estimate	OR (odds ratio)	*P* value	Effect estimate	Standard error	OR	*P* value
**Smoking**							
Current	−0.726	0.484	0.393				
Former	0.250	1.283	0.652				
None	ref						
**Caffeine**	−0.0013	0.999	0.449				
**Age of menarche**							
> 12	−0.475	0.622	0.334				
≤ 12	ref						
**Breastfeed**^**c**^^**d**^	0.765	2.148	0.066	0.899	0.402	2.457	**0.026**
**OCP duration**							
10+ years	−0.123	0.884	0.876				
5–10 years	−0.329	0.720	0.499				
2–5 years	0.330	1.391	0.729				
< 2 years	ref						
**CRP**^d^	0.321	1.378	0.046	0.344	0.169	1.411	**0.042**
**Ferritin**	−0.001	0.999	0.582				
**Osteoporosis**	−0.064	0.938	0.905				
**Osteoarthritis**	0.446	1.562	0.482				
**Hysterectomy**	−0.087	0.917	0.688				
**Oophorectomy**	−0.241	0.786	0.702				
**Salpingectomy**	−0.251	0.778	0.452				
**Fibroids**	−1.240	0.289	0.337				
**CAD**^d^	1.421	4.140	0.051	1.609	0.630	4.996	**0.011**
**HLD**	0.366	1.442	0.363				
**Non-ovarian cancer**	−1.040	0.354	0.159	−1.351	0.733	0.259	0.065

*EtOH* Alcohol, *CHC* Current hormonal contraception, *OCP* Oral contraceptive pill, *CRP* C reactive protein, *CAD* Coronary artery disease, *HLD* Hyperlipidemia

^a^ All univariable regressions were adjusted for baseline variables including age, race/ethnicity, BMI, parity and current hormonal replacement therapy (HRT) use

^b^ Multivariable regressions included the baseline variables (age, race/ethnicity, BMI, parity, HRT) and eligible variables from univariable analyses with cut-off *p* value < 0.25

^c^ For the “Breastfeed” variable, the reference for parity is one instead of none

^d^ These variables demonstrated significant associations with CA125 level ≥ 35 U/ml in the multivariable logistic regression model

## Discussion

This study analyzed associations between various demographic, clinical, and laboratory variables and CA125 levels in both premenopausal and postmenopausal women without history of OC in a large nationally representative sample. CA125 level has been reported to be higher in pre- versus postmenopausal women and decreases with advancing age [17, 26]. Our results were consistent. In agreement with prior studies, [17, 18] this study also found that CA125 levels were lower in non-White compared to White women. Study participants with higher BMI had lower CA125 concentration, consistent with findings from previous studies that obesity was associated with lower CA125 level, likely due to the dilution effect of high plasma volume [18, 27].

Of the reproductive/gynecologic history variables examined, hysterectomy history was not significantly associated with CA125 levels in the multivariable analyses in postmenopausal women, though the crude linear regression did show an inverse relationship (− 8.06%, *p* = 0.05), which is consistent with the results of prior studies suggesting that hysterectomy history may be associated with lower CA125 level [17, 18]. In premenopausal women, this study demonstrated that 5–10 years’ duration of OCP use, compared to duration < 2 years, was less likely to be associated with CA125 level ≥ 35 U/ml. Sasamoto et al. had shown that longer duration of OCP use (> 4 years), compared to duration < 2 years, was associated with lower CA125 level in premenopausal women without OC [19]. This finding correlates with the duration-dependent protective association between longer OCP use and risk reduction of OC by suppressing ovulation [28–30]. This study found that postmenopausal women with breastfeeding history were more likely to be associated with CA125 level ≥ 35 U/ml. To our knowledge, there has been no prior literature reporting on the direct relationship between breastfeeding and CA125 level. Breastfeeding has been reported to be associated with 24% decrease in risk of ovarian cancer; the protective mechanism is hypothesized to be the inhibition of epithelial cell in the setting of ovulation suppression during breastfeeding [31]. The association between breastfeeding and CA125 revealed in this study seems to be contrary to expectation, which may reflect a true association between breastfeeding history and CA125 level in women without OC, or may reflect bias from small sample size in the analysis (*N* = 23 who had CA125 level ≥ 35 U/ml in postmenopausal subgroup) or the choice of cut-off value for CA125.

Higher CA125 levels have been reported in women with active endometriosis, and CA125 was even proposed as a candidate screening biomarker for endometriosis [32, 33]. However, among postmenopausal women, history of endometriosis has been reported to be associated with lower CA125 level, [7, 9] likely reflecting the resolution of endometriosis after menopause or management. This study similarly demonstrated that in premenopausal women, self-reported diagnosis of endometriosis was associated with higher CA125 levels, although this association was not retained in multivariable linear or logistic analyses. This study also showed that in postmenopausal women, history of endometriosis was associated with lower CA125 levels in univariable linear regression. However, the significance disappeared in multivariable analysis, and logistic regressions could not be performed due to sample size limitation. One explanation is that in postmenopausal women, measurement of CA125 can reflect not only the presence/absence of a historical gynecologic diagnosis, but also the subsequent clinical management and current status of the condition, whether it remains active.

In terms of bone health, osteoporosis was significantly associated with lower CA125 levels in premenopausal women by crude linear regression (− 31.13%, *p* = 0.049) and showed similar trend in postmenopausal women via both univariable linear regression (− 1.69%, *p* = 0.750) and logistic regression (OR = 0.938, *p* = 0.905). This finding was consistent with previously reported positive correlation between bone mineral density and CA125 level in both pre- and postmenopausal women [34], reflecting a lower CA125 level in setting of hypoestrogenic state contributing to decreased bone density**.** For postmenopausal women, Akinwunmi et al. had demonstrated that both osteoporosis and osteoarthritis were associated with lower levels of CA125, [9] however our study did not reveal such significant associations (*N* = 76 for osteoporosis and *N* = 225 for osteoarthritis in Akinwunmi’s study, versus *N* = 203 for osteoporosis and *N* = 175 for osteoarthritis in this study).

Regarding cardiovascular comorbidities, this study showed decreased levels of CA125 in premenopausal women with CAD in crude linear regression (− 49.79%, *p* = 0.001). This finding contradicts Akinwunmi et al’s study in which CA125 was reported to be higher in premenopausal women with CAD based on a very small number of CAD cases (N = 2 versus *N* = 10 in present study) [9]. Given the rarity of CAD in young women, interpretation of the association between CA125 level and CAD in the premenopausal setting is difficult. For postmenopausal women, history of CAD was associated with higher CA125 levels in the multivariable linear regression model (28.27%, *p* = 0.047) and more likely to be associated with CA125 level ≥ 35 U/ml in multivariable logistic regression (OR = 5.00, *p* = 0.011); these findings are in agreement with prior literature [9, 35]. Other studies have suggested that increased CA125 may be associated with heart failure and pericardial effusions, hypothesizing that higher levels of CA125 are secreted from pericardium in the setting of mechanical stretch, irritation, and inflammation [36, 37]. The association between CAD and increased CA125 in postmenopausal women might be explained by the level of active or uncontrolled cardiac inflammation or decompensated heart failure at the particular cross-sectional time-point of this study.

To explore the relationship between inflammation and CA125 level, laboratory biomarkers of inflammation (CRP and ferritin) were included in this study. As a protein produced by coelomic epithelial cells lining the pleura, peritoneum and ovaries in the setting of inflammatory stress, CA125 level was hypothesized to demonstrate positive association with inflammatory biomarkers [9, 38]. The study showed that increasing CRP level correlated with higher levels of CA125 by crude linear regressions in both premenopausal and postmenopausal subgroups, and significantly increased the odds of having CA125 level ≥ 35 U/ml by multivariable logistic analysis in the postmenopausal subgroup. No association between ferritin level and CA125 level was demonstrated regardless of menopausal status.

History of non-ovarian cancer was found to be associated with higher CA125 levels in premenopausal women. In postmenopausal women, those with non-ovarian cancer history tended toward a lower likelihood of having CA125 levels ≥ 35 U/ml. Funston et al. have reported in a UK population-based study that 12.3% of those with CA125 level ≥ 35 U/ml were diagnosed with a non-ovarian cancer, and non-ovarian cancer diagnosis was found in 20.4% of women with CA125 level ≥ 35 U/ml who were ≥ 50 years of age [39].The results from our postmenopausal group deviated from such findings, perhaps reflecting some component of prior cancer-directed management and/or difference stemming from this study examining the US population. As noted, information regarding cancer diagnosis in this study was based on interview questions, and more specific details about the cancer diagnosis such as the time of diagnosis, whether or not the cancer is active, and treatment history could not be ascertained. Some of those who recorded a history of non-ovarian cancer in our study might have been cured a long time ago with CA125 levels falling within normal range at the time of study measurement. As such, the real association between non-ovarian cancer and CA125 level, especially in postmenopausal women, might be masked or diminished in this analysis. Moreover, this study did not examine the relationships between each specific non-ovarian cancer and CA125 level. Study participants reported history of major non-ovarian cancers including breast cancer (24%), non-melanoma skin cancer (20%), cervical cancer (14%), colon cancer (7%), lung cancer (3%), lymphoma (2%), stomach cancer (1%), and liver cancer (0.5%). Given the known strong association between breast cancer and higher CA125 levels as previously reported, [9] further investigation of breast cancer history and CA125 level was done, using crude linear regression. Breast cancer history was found to have a potentially marginal association with higher CA125 level in premenopausal women (*N* = 7, effect estimate = 0.63, *p* = 0.082) but did not reach any statistical significance in postmenopausal women (*N* = 41, *p* = 0.985).

Current smoking was associated with lower CA125 level among premenopausal women in both multivariable linear and logistic regressions, consistent with previous findings [17, 18]. Smoking has been postulated to potentiate hepatic enzymes which accelerate metabolic degradation of CA125 [17]. In postmenopausal women only, more than 2 drinks a day was associated with lower CA125 in crude linear regression. No previous studies have evaluated the relationship between alcohol consumption and CA125 level. Perhaps severe alcohol consumption may upregulate hepatic metabolism of CA125, resulting in its serum decline. This study also found that in postmenopausal women only, more caffeine intake was associated with lower CA125 level after adjusting for baseline confounding variables, which is consistent with Pauler et al’s findings that caffeine consumption decreased CA125 level [17].

This study has several unique strengths. First, study participants comprised a nationally representative sample from across the United States. Results from this data are more generalizable compared to results from control groups in previous studies, which were more restricted in terms of the ethnicities, ages and/or geographies represented. Furthermore, those study enrollees from long-term cancer screening or prevention clinical trials tended to be generally healthier compared to the general population [40]. Another strength is the inclusion of both premenopausal and postmenopausal women, allowing for clearer comparisons in two subgroups. Both linear regression and logistic regression methods facilitated multivariable adjustments, enabling confirmation of significant associations between CA125 level and multiple candidate factors, and increasing the precision of analyses from these different dimensions. The linear regressions could be used to evaluate trends over time either for potential screening for early detection of OC or disease surveillance and monitoring response to therapy in patients with OC. The dichotomized method utilizing a cutoff value to define abnormally elevated CA125 generates more data for an approach to interpreting CA125 results as a clinical screening biomarker in a previously unscreened population. Moreover, this study examined further the relationship between CAD and CA125 level, which has not been explored in depth in existing literature. Associations between history of CAD with CA125 levels stratified by menopausal status in this study differed from those previously published in literature, suggesting the need to re-examine reported associations given limitations in sample sizes of studies to date. Finally, this is the one of the first few studies to examine the relationship between clinical biomarkers of inflammation and CA125 levels.

We acknowledge several limitations of this study. The cross-sectional design did not allow for longitudinal measurements of CA125 and precluded interpretation of CA125 trends over time. Study participants provided only a snapshot of their clinical history and lifestyle practices at one particular point in time via self-report without further validation. The clinical data obtained regarding participants' medical conditions, especially with respect to the non-ovarian cancer diagnosis, may not have reflected the actual status of such medical conditions. Whether a participant was in remission, receiving treatment, or progressing at the moment when CA125 level was measured, and how long ago the treatment was given, cannot be confirmed. As such, this analysis was unable to eliminate the confounding effect related to the status of non-ovarian cancer diagnoses and other medical conditions, and the real associations might be underestimated. It is interesting to note, however, that Akinwunmi et al. previously found no evidence that treatments associated with a particular medical condition changed the association between the medical condition and CA125 level [9]. Additionally, this study does not include all the possible factors that may affect CA125 levels, so these results may be biased by unmeasured or unknown confounders. For instance, *BRCA* mutation status and family history of OC could not be assessed, although increased CA125 level has been associated with *BRCA* mutations and familial OC [5]. Other factors, such as history of heart failure, cirrhosis, and the specific menstrual cycle phase of each study participant at time of survey, which have been reported to impact CA125 levels could not be included here [9, 41–43]. Some potential correlating factors, such as the use of intrauterine device with hormones, history of pelvic inflammatory disease and polycystic ovary syndrome, were not included in the questionnaire of NHANES 2001–2002. Additionally, under-diagnosis of benign gynecologic diseases such as uterine fibroids and endometriosis is common, especially in asymptomatic patients, and may contribute to masking certain associations. Furthermore, the choice of cut-off value for CA125 would potentially impact the study results. We used 35 U/ml as a cutoff based on recommendations of National Institute for Health and Care Excellence (NICE); this cutoff was derived from a prior study in which only 1% of apparently healthy persons, 6% of patients with nonmalignant disease, and 82% of patients with surgically demonstrated ovarian cancer had a CA125 level ≥ 35 U/ml [2, 25]. Funston et al. has reported that a cut-off value of 23 U/ml provided a higher sensitivity but lower specificity (86.4% and 86%) compared to standard cut-off (35 U/ml) (78.5% and 94.5%) for ovarian cancer diagnosis, in a retrospective cohort study examining routinely collected primary care and cancer registry data from the UK [44]. By performing logistic regressions based on this new cut-off (433 participants with CA125 level ≥ 23 U/ml), comparable findings were demonstrated, consistent with that generated from using the cutoff of 35 U/ml. In premenopausal women, longer OCP use and current use of CHC were more likely to be associated with CA125 level ≥ 23 U/ml by multivariable logistic regression; history of endometriosis was associated with CA125 level ≥ 23 U/ml in univariable logistic regression only. In postmenopausal women, relatively similar associations were found in crude analyses, especially that elevated CRP level significantly increased the odds of having CA125 level ≥ 23 U/ml; however, none of these potential variables reached significance in multivariable regression analyses (see supplementary tables). Similar exposure factors could lead to fluctuations in CA125 levels when 23 U/ml was applied as the cut-off, supplying a rationale for the reduced specificity of 23 U/ml as the cut-off for an elevated CA125 level in Funston et al’s study. Finally, these results were based on sampling participants in 2001–2002, and may no longer be reflective of current associations between CA125 levels and various candidate correlating factors, given changes in disease demographics, management, and lifestyle practices over time.

## Conclusion

In summary, this is a large analysis of factors associated with CA125 levels among women without OC, and a broad population-level study of CA125 levels in both pre- and postmenopausal women across the United States. In premenopausal women, CA125 was found to be lower in current smokers and OCP users of moderately long duration, but higher in those with non-ovarian cancer. In postmenopausal women, CA125 was found to be higher in those with CAD, breastfeeding history, and elevated CRP level. Such associations should be evaluated and weighed in routine clinical interpretation of CA125 levels. Future studies examining underlying biologic and pathologic mechanisms of the observed associations are warranted. Studies with larger sample sizes are needed to improve statistical power, distill more robust findings and refine a strategy for CA125 interpretation in individual patients.

## Supplementary Information

**Additional file 1.**

## Data Availability

The datasets used and analyzed during the current study are available in National Health and Nutrition Examination Survey (NHANES) 2001–2002 repository. (https://wwwn.cdc.gov/nchs/nhanes/continuousnhanes/default.aspx?BeginYear=2001).

## Abbreviations

CA125: Cancer antigen 125
OC: Ovarian cancer
OCP: oral contraceptive pill
HRT: Hormone replacement therapy
NHANES: National Health and Nutrition Examination Survey
CV: Coefficient of variation
BMI: Body mass index
CHC: Current hormonal contraception
CAD: Coronary artery disease
CRP: C reactive protein
SD: Standard deviation
CI: Confidence interval
EtOH: Alcohol
HLD: Hyperlipidemia
OR: Odds ratio
NICE: National Institute for Health and Care Excellence
