# Supplementary material for: Factors associated with serum CA125 level in women without ovarian cancer in the United States: a population-based study
Source: BMC Cancer. 2022 May 14;22:544. doi: 10.1186/s12885-022-09637-7 (PMC9107191; doi:10.1186/s12885-022-09637-7)
Supplement: Supplementary file 1 — Additional file 1. [file 12885_2022_9637_MOESM1_ESM.docx]

**Supplementary Table 1.** Associations between candidate correlating factors and CA125 level ≥ 35 U/ml versus CA125 level ≥ 23 U/ml (results shown in red, only odds ratio and p-values are shown here) among premenopausal women (N=1157) by univariable and multivariable logistic regressions

| **Variables** | | **Univariable logistic regressions** | | |  | **Multivariable logistic regressions** | | | | | |
| --- | --- | --- | --- | --- | --- | --- | --- | --- | --- | --- | --- |
|  | Effect estimate | | Odds ratio (OR) | P value | Effect estimate | | Standard  error | OR | | P value | |
| **Smoking** |  | |  |  |  | |  |  | |  | |
| Current | -0.654 | | 0.520 (0.792) | 0.0893 (0.402) | -0.863 | | 0.426 | 0.422 | | **0.043** | |
| Former | 0.311 | | 1.365 (0.942) | 0.471 (0.786) | 0.579 | | 0.400 | 1.784 | | 0.148 | |
| None | ref | |  |  |  | |  |  | |  | |
| **EtOH** |  | |  |  |  | |  |  | |  | |
| >2 drinks/day | -0.165 | | 0.848 (0.799) | 0.679 (0.523) |  | |  | (0.235) | | (0.055) | |
| 1-2 drinks/day | 0.092 | | 1.096 (0.711) | 0.763 (0.205) |  | |  | (0.323) | | (0.096) | |
| None | ref | |  |  |  | |  |  | |  | |
| **Caffeine** | -0.00037 | | 1.000 (1.000) | 0.530 (0.608) |  | |  |  | |  | |
| **Age of menarche** |  | |  |  |  | |  |  | |  | |
| >12 | 0.118 | | 1.125 (1.195) | 0.634 (0.256) |  | |  |  | |  | |
| ≤12 | ref | |  |  |  | |  |  | |  | |
| **Breastfeed** | 0.012 | | 1.012 (0.882) | 0.968 (0.493) |  | |  |  | |  | |
| **CHC use** | -1.019 | | 0.361 (0.202) | 0.570 (0.027) |  | |  | (0.178) | | **(0.038)** | |
| **OCP duration** |  | |  |  |  | |  |  | |  | |
| 10+ years | -0.483 | | 0.617 (0.405) | 0.407 (0.024) | -0.596 | | 0.621 | 0.551  (0.249) | | 0.337  **(0.035)** | |
| 5-10 years***** | -0.967 | | 0.380 (0.919) | 0.098 (0.821) | -1.173 | | 0.548 | 0.309  (0.688) | | **0.032**  (0.757) | |
| 2-5 years | 0.528 | | 1.695 (1.551) | 0.234 (0.199) | 0.465 | | 0.474 | 1.592  (0.823) | | 0.327  (0.798) | |
| <2 years | ref | |  |  |  | |  |  | |  | |
| **CRP** | 0.325 | | 1.383 (1.168) | 0.154 (0.301) | 0.295 | | 0.260 | 1.343 | | 0.256 | |
| **Ferritin** | -0.00195 | | 0.998 (1.000) | 0.621 (0.775) |  | |  |  | |  | |
| **Oophorectomy** | -0.331 | | 0.718 (0.929) | 0.662 (0.907) |  | |  |  | |  | |
| **Salpingectomy** | -0.616 | | 0.540 (0.698) | 0.278 (0.213) |  | |  | (1.440) | | (0.609) | |
| **Endometriosis** | 0.857 | | 2.355 (3.757) | 0.029 (0.003) | 0.763 | | 0.631 | 2.145  (3.081) | | 0.226  (0.181) | |
| **Fibroids** | 0.456 | | 1. 578 (2.135) | 0.468 (0.066) |  | |  | (NA) | | (NA) | |
| **HLD** | -0.412 | | 0.662 (1.145) | 0.480 (0.615) |  | |  |  | |  | |
| **Non-ovarian cancer** | 0.058 | | 1.060 (1.598) | 0.938 (0.451) | | |  |  |  | |  |

**Supplementary Table 2.** Associations between candidate correlating factors and CA125 level ≥ 35 U/ml versus CA125 level ≥ 23 U/ml (results shown in red, only odds ratio and p-values are shown here) among postmenopausal women (N=1116) by univariable and multivariable logistic regressions

| **Variables** | **Univariable logistic regressions** | | | **Multivariable logistic regressions** | | | |
| --- | --- | --- | --- | --- | --- | --- | --- |
|  | Effect estimate | OR (odds ratio) | P value | Effect estimate | Standard error | OR | P value |
| **Smoking** |  |  |  |  |  |  |  |
| Current | -0.726 | 0.484 (1.299) | 0.393 (0.410) |  |  |  |  |
| Former | 0.250 | 1.283 (1.058) | 0.652 (0.837) |  |  |  |  |
| None | ref |  |  |  |  |  |  |
| **Caffeine** | -0.0013 | 0.999  (0.999) | 0.449  (0.125) |  |  | (1.000) | (0.619) |
| **Age of menarche** |  |  |  |  |  |  |  |
| >12 | -0.475 | 0.622 (0.995) | 0.334  (0.986) |  |  |  |  |
| ≤12 | ref |  |  |  |  |  |  |
| **Breastfeed** | 0.765 | 2.148 (0.905) | 0.066 (0.740) | 0.899 | 0.402 | 2.457 | **0.026** |
| **OCP duration** |  |  |  |  |  |  |  |
| 10+ years | -0.123 | 0.884  (1.073) | 0.876 (0.915) |  |  |  |  |
| 5-10 years | -0.329 | 0.720  (1.193) | 0.499  (0.702) |  |  |  |  |
| 2-5 years | 0.330 | 1.391  (1.418) | 0.729  (0.510) |  |  |  |  |
| <2 years | ref |  |  |  |  |  |  |
| **CRP** | 0.321 | 1.378  (1.325) | 0.046  (0.010) | 0.344 | 0.169 | 1.411  (1.238) | **0.042**  (0.447) |
| **Ferritin** | -0.001 | 0.999  (0.999) | 0.582  (0.509) |  |  |  |  |
| **Osteoporosis** | -0.064 | 0.938 (0.701) | 0.905  (0.060) |  |  |  |  |
| **Osteoarthritis** | 0.446 | 1.562  (0.993) | 0.482  (0.972) |  |  |  |  |
| **Hysterectomy** | -0.087 | 0.917  (0.887) | 0.688  (0.535) |  |  |  |  |
| **Oophorectomy** | -0.241 | 0.786  (0.985) | 0.702  (0.958) |  |  |  |  |
| **Salpingectomy** | -0.251 | 0.778  (1.676) | 0.452  (0.019) |  |  | (0.779) | (0.533) |
| **Fibroids** | -1.240 | 0.289  (0.364) | 0.337  (0.105) |  |  | (0.386) | (0.099) |
| **CAD** | 1.421 | 4.140  (1.493) | 0.051  (0.297) | 1.609 | 0.630 | 4.996 | **0.011** |
| **HLD** | 0.366 | 1.442  (1.116) | 0.363  (0.620) |  |  |  |  |
| **Non-ovarian cancer** | -1.040 | 0.354  (0.507) | 0.159  (0.106) | -1.351 | 0.733 | 0.259  (0.377) | 0.065  (0.276) |
